# Intensive chemotherapy for high-risk acute lymphoblastic leukemia in first remission: results from the NOPHO ALL2008 study

**DOI:** 10.1038/s41375-025-02789-y

**Published:** 2025-10-27

**Authors:** Atte Nikkilä, Olli Lohi, Jonas Abrahamsson, Laimonas Griskevicius, Helene Hallböök, Ólafur Gísli Jónsson, Bendik Lund, Hanne Vibeke Marquart, Ulrik Overgaard, Katrin Palk, Kaie Pruunsild, Petter Quist-Paulsen, Nina Toft, Goda Elizabeta Vaitkeviciene, Ulla Wartiovaara-Kautto, Mats Heyman, Kim Vettenranta, Kjeld Schmiegelow

**Affiliations:** 1 Tampere Center for Child Health Research, Tampere University, Tampere, Finland; 2 Tays Cancer Center, Tampere University Hospital, Tampere, Finland; 3 Department of Pediatrics, Institute of Clinical Sciences, Sahlgrenska Academy at University of Gothenburg, Gothenburg, Sweden; 4 Hematology, Oncology and Transfusion Medicine Center, Vilnius University Hospital Santaros Klinikos and Vilnius University, Vilnius, Lithuania; 5 Department of Medical Sciences, Uppsala University, Uppsala, Sweden; 6 Department of Pediatrics, Landspitali University Hospital, Reykjavík, Iceland; 7 Department of Pediatrics, St. Olav’s Hospital, Trondheim University Hospital, Trondheim, Norway; 8 Department of Clinical Immunology, Copenhagen University Hospital (rigshospitalet), Copenhagen, Denmark; 9 Institute of Clinical Medicine, Faculty of Medicine, University of Copenhagen, Copenhagen, Denmark; 10 Department of Hematology. National Hospital, Copenhagen, Denmark; 11 Department of Hematology, North Estonia Medical Centre, Tallinn, Estonia; 12 Department of Onco-haematology, Talinn Children’s Hospital, Talinn, Estonia; 13 Department of Hematology, St. Olav’s Hospital, Trondheim University Hospital, Trondheim, Norway; 14 Department of Hematology, Rigshospitalet University Hospital, Copenhagen, Denmark; 15 Center for Pediatric Oncology and Hematology, Institute of Clinical Medicine, Faculty of Medicine, Vilnius University, Vilnius, Lithuania; 16 Department of Hematology, Helsinki University Hospital Comprehensive Cancer Center, University of Helsinki, Helsinki, Finland; 17 Applied Tumor Genomics Research Program, Faculty of Medicine, University of Helsinki, Helsinki, Finland; 18 Childhood Cancer Research Unit, Karolinska Institute, Astrid Lindgren’s Children’s Hospital, Karolinska University Hospital, Stockholm, Sweden; 19 University of Helsinki and Hospital for Children and Adolescents, University of Helsinki, Helsinki, Finland; 20 Department of Pediatrics and Adolescent Medicine, Rigshospitalet University Hospital, Copenhagen, Denmark

**Keywords:** Acute lymphocytic leukaemia, Paediatrics

Survival rates have improved markedly for childhood acute lymphoblastic leukemia (ALL) across age groups [1, 2], but less so for high-risk (HR) cases. Previous studies have reported five-year event-free survival (EFS) rates ranging from 50.1% to 75.3% for this subgroup [3–5]. In a subgroup analysis of the earlier NOPHO ALL-92 and ALL-2000 protocols, most patients with T-cell (T-ALL) and/or white blood cell count (WBC) over 100 × 10⁹/l combined with >25% leukemic blasts on day 15 in the bone marrow showed poor outcomes, with five-year EFS below 50% [4]. The HR arm of the NOPHO ALL2008 protocol was designed to include patients aged 1– ≤ 45-years with the poorest prognoses and investigate whether intensified chemotherapy could raise EFS to over 60% [2, 4, 6, 7]. Frontline therapy included a three-drug induction using either prednisolone (B-cell precursor ALL (BCP-ALL) with WBC < 100 **×** 10^9^/l) or dexamethasone (BCP-ALL with WBC ≥ 100 **×** 10^9^/l or T-cell ALL), after which patients were stratified into three arms: standard risk (SR), intermediate risk (IR) or HR **(**Supplementary Table 1**)** [2]. Patients were allocated to the HR chemotherapy arm if they had (i) hypodiploidy (<45 Chromosomes and/or DNA index <0.85) or *KMT2A*-rearranged (*KMT2A*-r), (ii) T-lineage ALL and/or WBC ≥ 100 **×** 10^9^/l and ≥25% minimal residual disease (MRD) on day 15 or ≥0.1% on day 29 (end of induction, EOI), (iii) any patient with MRD ≥ 5% at EOI, or (iv) ≥0.1% MRD on day 79 (end of consolidation, EOC) for patients who were initially stratified as SR or IR. MRD assessment for BCP-ALL was based on response assessment by flow cytometry, while polymerase chain reaction (PCR) was used for T-ALL; in case of missing markers, the other modality could be used. Patients who were assigned to receive hematopoietic stem cell transplantation (HSCT) (EOI MRD ≥ 5%, EOC MRD ≥ 0.1%, post-block-B1 ≥ 0.1%) were included in the analysis and they were censored two weeks prior to transplantation. Survival and relapse rates are reported as 5-year estimates unless otherwise specified. For more details, see the Materials and Methods section in the Supplementary Data.

Out of 1719 patients enrolled in the NOPHO ALL2008 protocol, 314 were allocated to the HR arm (Supplementary Fig. 1). Among these, 75% (*N* = 234) were HR due to poor treatment response, while the remaining 25% (*N* = 80) were identified based on predefined high-risk genetic features (*KMT2A*-r or hypodiploidy). The age range of the HR cohort was 1.0 to 44.9 years, with a median age of 11.9 years - substantially higher than the median age of the entire NOPHO ALL2008 cohort (5.5 years) [2] (Table 1).

**Table 1:** The characteristics of patients in the high risk arms (chemotherapy and HSCT) of NOPHO ALL2008 stratified by the induction therapy.

	All patients	Prednisolone	Dexamethasone
N	314	122	192
Sex			
Male	188 *(60%)*	67 *(55%)*	121 *(63%)*
Female	126 *(40%)*	55 *(45%)*	71 *(37%)*
Age (years)			
1 - <10	140 *(45%)*	56 *(46%)*	84 *(44%)*
≥10 - <18	82 *(26%)*	30 *(25%)*	52 *(27%)*
≥18	92 *(29%)*	36 *(30%)*	56 *(29%)*
Cell lineage			
B	185 *(59%)*	121 *(99%)*	*64 (33%)*
T	129 *(41%)*	1 *(1%)*	128 *(67%)*
WBC (10^9^/l)			
< 100	194 *(62%)*	120 *(98%)*	74 *(39%)*
≥ 100	120 *(38%)*	2 *(2%)*	118 *(61%)*
t(12;21)			
Yes	*5 (2%)*	*5 (4%)*	*0 (0%)*
No	309 *(98%)*	117 *(96%)*	192 *(100%)*
High hyperdiploidy			
Yes	20 *(6%)*	*13 (11%)*	7 *(4%)*
No	294 *(94%)*	*109 (89%)*	185 *(96%)*
Hypodiploid			
Yes	19 *(6%)*	13 *(11%)*	6 *(3%)*
No	295 *(*94*%)*	109 *(89%)*	186 *(97%)*
*KMT2A*-r			
Yes	54 *(17%)*	20 *(16%)*	34 *(18%)*
No	260 *(83%)*	102 *(84%)*	158 *(82%)*
Median age at diagnosis (y)	11.9	11.6	12.3
Median WBC (10^9^/l)	46.1	9.50	127
Stem cell transplantation	124 *(39%)*	74 *(61%)*	50 *(26%)*

*WBC* white blood cell count, *KMT2A*-r *KMT2A*-rearranged.

After censoring patients two weeks prior to HSCT, the EFS was 64% (95% confidence interval (CI), 57% to 70%) and overall survival (OS) 70% (95% CI, 64% to 76%) (Fig. 1A, B). The relapse rate (RR), with death in complete remission (CR) as a competing event, was 24% (95% CI, 18% to 30%) (Fig. 1A). Without censoring for HSCT, the observed EFS was 66% (95% CI, 61% to 71%) and OS 71% (95% CI, 66% to 76%).

**Fig. 1:**
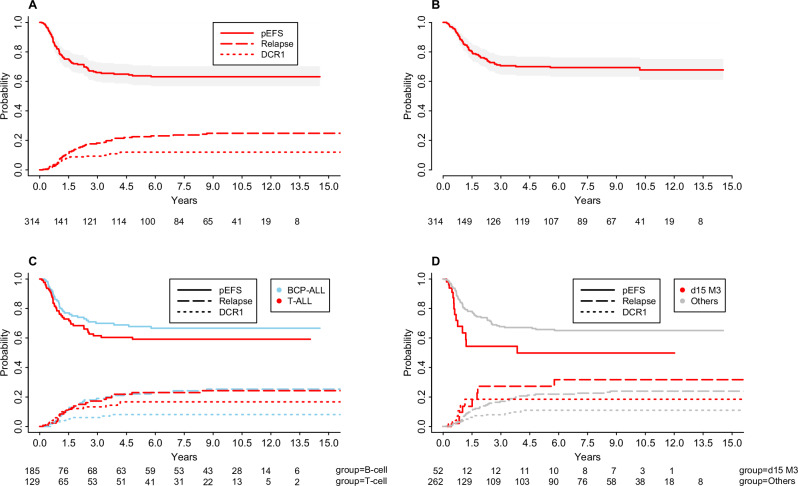
The HR cohort’s overall and event-free survival curves, along with the cumulative incidences of relapse and death. The number of participants at risk is provided at the bottom of each panel. **A** Event-free survival and cumulative incidence of death and relapse with 95% confidence intervals. **B** Overall survival with 95% confidence intervals. **C** Event-free survival and cumulative incidence of death and relapse by cell lineage. **D** Event-free survival and cumulative incidence of death and relapse for patients stratified to block therapy on day 15.

All subjects achieved morphological CR either during induction (66%), after block A1 (27%), block B1 (5%), or block C1 (2%). After block A1, 71% of patients (196 out of 276 with MRD data) achieved MRD levels below 0.1% (Supplementary Fig. 2**)**. This proportion increased to 86% (236 out of 274) and 92% (216 out of 235) following blocks B1 and C1, respectively. Of those who proceeded to block B1, 14% (38 out of 276) had MRD ≥ 0.1% after block A1 and were assigned to receive HSCT [8].

Patients with BCP-ALL (*N* = 185) had an EFS of 68% (95% CI, 59% to 77%) with RR of 24% (95% CI, 16% to 32%), while patients with T-ALL (*N* = 129) had an EFS of 59% (95% CI, 49% to 69%) and RR of 23% (95% CI, 14% to 32%) (Fig. 1C). Treatment-related mortality in first remission (DCR1) was 8% in BCP-ALL compared to 17% in T-ALL (*p* = 0.05) (Fig. 1C).

Patients aged above 18 years had an EFS of 53% (95% CI, 39% to 67%) and the two younger patient groups (0–8.99 y and 9-17.99 y) had EFSs of 67% (95% CI, 58% to 77%) and 68% (95% CI, 55% to 81%), respectively (Log-rank *p* = 0.12) (Supplementary Figure 3A). RR was 32% (95% CI, 19% to 46%) in patients over 18 years, whereas younger patient groups had respective RRs of 23% (CI 15 to 32%) and 15% (CI 5 to 25%). The DCR1 rate was 12% (95% CI, 3% to 21%) in patients above 18 years and for the younger groups 9% (95% CI, 4% to 15%) and 17% (95% CI, 7% to 27%), respectively.

Among patients stratified to the HR arm due to inadequate treatment response, the EFS after censoring for HSCT was 58% (95% CI, 50% to 67%), and the RR was 29% (95% CI, 21% to 37%) (Supplementary Fig. 3B). For those stratified based on protocol-defined HR genetics, the EFS was 74% (95% CI, 63% to 84%) and the RR was 13% (95% CI, 5% to 21%).

*KMT2A*-r was identified in 54 patients (17% of the HR cohort), including eight patients with T-ALL. After censoring for HSCT, the EFS and OS were 76% (95% CI, 64% to 88%) and 80% (95% CI, 69% to 92%), respectively, with RR of 16% (95% CI, 5% to 26%). When stratified by EOI response, the difference did not reach statistical significance (EOI MRD < 0.1% EFS: 80%, 95% CI 64% to 96%, EOI MRD ≥ 0.1% EFS: 74%, 95% CI 52% to 97%) (Supplementary Fig. 3C).

Hypodiploidy was identified in 19 patients (6% of the HR cohort). Among these, 53% (*N* = 10) achieved MRD < 0.1% at EOI, 26% (*N* = 5) had undetectable MRD, and three patients had no MRD data available. After censoring for HSCT patients, the EFS and OS for patients with hypodiploidy were both 67% (95% CI, 44% to 91%), with non-relapse events being most frequent. DCR1 occurred in 3 patients, and 1 patient developed a second malignant neoplasm (SMN). With death in CR as a competing event, the RR was 7% (95% CI, 0% to 20%) (Supplementary Fig. 3D).

Notably, all patients with centrally confirmed *KMT2A*-r (*n* = 13) or hypodiploidy (*n* = 5) and undetectable MRD at EOI remained relapse-free. Additionally, none of the 17 patients with *KMT2A*-r who had WBC counts below 100 × 10⁹/l at diagnosis and MRD levels below 0.1% at EOI experienced relapse.

According to protocol, induction therapy was discontinued for 52 patients (75% male) on day 15 due to M3 marrow, and they were immediately shifted to block-type therapy. Among them, 24 patients remained in CR, 18 relapsed, 9 died in remission, and one developed a SMN. Median WBC count and age did not differ significantly between patients in CR (126 × 10⁹/l; 7.7 years) and those who relapsed (148 × 10⁹/l; 16.6 years) (*p* = 0.74 and *p* = 0.46, respectively). The EFS after censoring for HSCT was 50% (95% CI, 30% to 70%), with a relapse rate (RR) of 32% (95% CI, 13% to 51%) (Fig. 1D). Patients who continued with induction therapy had an EFS of 60% (95% CI, 38% to 82%) and a RR of 27% (95% CI, 6% to 47%).

In multivariate modeling, high WBC and age ≥18 years were associated with increased hazards for EFS, with HR of 2.80 (95% CI, 1.42 to 5.51) and 2.33 (95% CI, 1.26 to 4.30), respectively (Supplementary Table 2). Doubling of WBC count increased the hazard by 1.18 (95% CI, 1.07 to 1.31), and by 1.26 (95% CI, 1.09 to 1.44) when modeled as a continuous variable.

In summary, the NOPHO ALL2008 protocol achieved the main goal of minimizing the number of patients assigned to intensive chemotherapy, with only 18% of the entire cohort allocated to the HR arm, representing a significant reduction compared to earlier NOPHO protocols [4]. The estimated EFS and OS align with those from international studies despite differences in patient selection and HSCT use [9–14]. Adjusted Cox models confirmed the established prognostic markers of poor outcome in this patient population, namely high WBC counts and adult age. While the poor prognosis associated with *KMT2A*-r in infants is well established, our data indicate that patients over one year of age treated with NOPHO ALL2008-type HR chemotherapy had reasonably favorable outcomes. Similarly, hypodiploid ALL patients who did not undergo HSCT demonstrated relatively good outcomes in our cohort. Notably, most adverse events in this group were related to treatment toxicity rather than disease progression, arguing against further intensification of therapy for hypodiploid ALL.

Key limitations of this study include the lack of centralized cytogenetic and MRD assessments, as well as the absence of course-specific toxicity reporting, which limited our ability to evaluate adverse events in relation to specific chemotherapy blocks. Nonetheless, central findings have informed the design of the ongoing semi-European ALLTogether trial, where the use of NOPHO ALL2008-type HR blocks is reserved for approximately 3% of patients with the poorest predicted prognosis. Looking ahead, the incorporation of novel immunotherapies holds promise for improving outcomes and reducing treatment-related toxicity in HR patients.

## Supplementary information

Supplementary material

## Data Availability

The datasets generated during and/or analysed during the current study are not publicly available due to restrictions related to participant and protocol-wide study permissions but are available from the corresponding authors on reasonable request and with permissions from involved countries and institutions.
