# Supplementary material for: Intensive chemotherapy for high-risk acute lymphoblastic leukemia in first remission: results from the NOPHO ALL2008 study
Source: Leukemia. 2025 Oct 27;40(1):215–8. doi: 10.1038/s41375-025-02789-y (PMC12789026; doi:10.1038/s41375-025-02789-y)
Supplement: Supplementary file 1 — Supplementary material [file 41375_2025_2789_MOESM1_ESM.pdf]

Supplementary material for manuscript:

”Intensive chemotherapy for high-risk acute lymphoblastic leukemia in first remission: results from the NOPHO ALL2008 study”

Supplementary Patients and Methods

The treatment schedule and overall results of the NOPHO ALL2008 protocol have been published previously. Briefly, all newly diagnosed ALL patients aged 1–≤45-years were enrolled in the participating countries. The exclusion criteria were Down syndrome, drug hypersensitivity, over one week of glucocorticoids before start of induction, previous malignancy, fertile females not accepting contraception, clinician's or principal investigator's decision, Ph chromosome positivity, and other lineages than B-, T- or bilineage ALL. The risk grouping for BCP-ALL was based on response assessment by flow cytometry, while PCR was used for T-ALL. In case of missing markers, the other modality could be used. Negative MRD was defined as being flow and PCR negative when both were available. According to the NOPHO ALL2008 protocol BCP-ALL should primarily be stratified using MRD-flow and T-ALL primary using PCR-MRD if informative markers are available. The quantitative range for the MRD methods used was between 0.1%–0.01% whereas the sensitivity was usually down to 0.01% or even lower for flow as well as PCR MRD. Age of the subjects did not affect treatment stratification.

High risk chemotherapy

Frontline therapy in the NOPHO ALL2008 protocol included a three-drug induction using either prednisolone (BCP-ALL with WBC <100 × 109/l) or dexamethasone (BCP-ALL with WBC >100 × 109/l or T-cell ALL), after which they were stratified into three arms: standard, intermediate or high-risk.

Patients were allocated to the HR chemotherapy arm if they had i) hypodiploidy (<45 Chrs and/or DNA index <0.85) or KMT2A-rearrangement; ii) T-lineage ALL and/or WBC ≥100 × 109/l and ≥25% MRD on day 15 or ≥0.1% on day 29 (EOI), iii) any patient with MRD ≥5% at EOI, or iv) ≥0.1% MRD on day 79 (end of consolidation , EOC) for patients who were initially stratified as SR or IR. Patients were eligible for HSCT in first complete remission (CR, MRD <5%) if they had ≥5% MRD on day 29 or after the first HR block if received block A on day 15, or ≥0.1% on day 79 or after the second HR block. The clinicians had an option to transplant adult patients with KMT2A-rearrangement or hypodiploidy in first CR. Patients who were assigned to receive HSCT were included in the analyses until they were censored two weeks prior to transplantation. Primary survival analysis by genetic stratification was based on the data as reported by the centers and includes some

erroneously classified cases (intention-to-treat); the manually scrutinized genetic data was used for subgroup analysis elsewhere.

Patients received a total of nine block courses (A1+B1+C1, A2+B2+C2, A3+B3+C3); however, for those with post-A1 MRD <0.1%, blocks C2 and C3 were omitted since November 2011 (174 / 55% of patients).

The intensive block-type chemotherapy was followed by a 60-week interim 6-mercaptopurine/methotrexate maintenance phase that included three high-dose methotrexate infusions (5 g/m2/24 hours with Leucovorin rescue from hour 42) and alternating intrathecal methotrexate or triple medication (prednisolone, cytarabine, methotrexate) at 6-week intervals. This was followed by a delayed intensification phase and classical maintenance therapy with intrathecal chemoprophylaxis at 8-week intervals until 2.5 years from the diagnosis. The treatment protocol did not include cranial irradiation for any patients. The efficacy and toxicity of liposomal cytarabine was explored by randomizing a total of 40 subjects to receive either conventional triple intrathecal therapy (cytarabine, methotrexate and hydrocortisone) or liposomal cytarabine.

Statistics

The last follow-up date for patients was 10th April 2024. Survival was estimated by the Kaplan-Meier method and differences tested by a two-sided log-rank test. Survival and relapse rate are reported as 5-year values throughout unless otherwise specified. For EFS, induction death, death in remission, relapse, or second malignancy were counted as events. No subject had events between their diagnosis and stratification to HR arm. Patients that received HSCT were censored two weeks before transplantation. The Cox proportional hazard model was applied to estimate time-dependent hazards of individual covariates; the proportionality assumption was assessed from Schoenfeld residuals. The cumulative incidence (CI) of relapse and death were calculated by using the Fine-Gray model (R package cmprsk), where death in CR was treated as a competing event. Linear models were fitted with 95% confidence intervals. All statistical tests were two-sided and $p<0.05$ was considered as statistically significant. The statistical analyses were carried out using R v. 3.5.2 (R Foundation for Statistical Computing, Vienna, Austria).

Ethics statement

The NOPHO ALL2008 protocol was approved by the ethics committees of treating cancer centers or national committees. Informed consent was obtained from all subjects/guardians according to the principles of Declaration of Helsinki.

Supplementary Table 1. Overview of the chemotherapy used in NOPHO ALL-2008

Block	Medication	Dose	Administration	Timing
Prednisolone induction	Prednisolone	60 mg/m^2/d	p.o.	d1-28, then taper in 9 days
	Vincristine	2.0 mg/m^2 (max 2.5 mg)	i.v.	d1, 8, 15, 22, 29
	Doxorubicin	40 mg/m^2	i.v.	d1, 22
	Methotrexate* (it-triple in CNS3/TLP)	8-12 mg (by age)	i.t.	d1, 8, 15, 29
Dexamethasone induction	Dexamethasone	10 mg/m^2/d	p.o.	d1-28, then taper in 9 days
	Vincristine	2.0 mg/m^2 (max 2.5 mg)	i.v.	d1, 8, 15, 22, 29
	Doxorubicin	40 mg/m^2	i.v.	d1, 22
	Methotrexate* (it-triple in CNS3/TLP)	8-12 mg (by age)	i.t.	d1, 8, 15, 29
Block A	Methotrexate	8-12 mg (by age)	i.t.	d1
	Cytarabine	20-30 mg (by age)	i.t.	d1
	Prednisolone	12.5-16 mg (by age)	i.t.	d1
	Cyclophosphamide	440 mg/m^2/d	i.v.	d1-5
	Etoposide	100 mg/m^2/d	i.v.	d1-5
	PEG-asparginase	1000 U/m^2/d	i.m./i.v.	d6
Block B	Methotrexate	8-12 mg (by age)	i.t.	d1
	Cytarabine	20-30 mg (by age)	i.t.	d1
	Prednisolone	12.5-16 mg (by age)	i.t.	d1
	Dexamethasone	20 mg/m^2/d	p.o.	d1-5
	6-mercaptopurine	10 mg/m^2/d	p.o.	d1-5
	Vincristine	2 mg/m^2 (max. 2.5 mg)	i.v.	d1, 6
	HD-Cytarabine	2 x 2 g/m^2/d	i.v.	d5-6
	HD-Methotrexate	5 g/m^2/24h	i.v.	d1
	PEG-asparginase	1000 U/m^2/d	i.m./i.v.	d6
Block C	Methotrexate	8-12 mg (by age)	i.t.	d1
	Cytarabine	20-30 mg (by age)	i.t.	d1
	Prednisolone	12.5-16 mg (by age)	i.t.	d1
	Fludarabine	30 mg/m^2/d	i.v.	d2-6
	Cytarabine	2 g/m^2/d	i.v.	d2-6
	Idarubicin	8 mg/m^2	i.v.	d2-6
	PEG-asparginase	1000 U/m^2/d	i.m./i.v.	d7
Maintenance 1	Methotrexate	20 mg/m^2/week	p.o	weekly
	6-mercaptopurine	75 mg/m^2/d	p.o.	daily
	Triple i.t.	See above	i.t.	12 weeks interval
	HD-methotrexate	5 g/m^2/d	i.v.	weeks 42, 66, 90
Delayed intensification	Dexamethasone	10 mg/m^2/d	p.o.	weeks 99, 101, then taper
	Vincristine	2.0 mg/m^2/d (max 2.5 mg)	i.v.	weeks 99, 100, 101, 102
	PEG-asparginase	1000 U/m^2/d	i.m./i.v.	week 99, 101
	Cyclophosphamide	1000 mg/m^2	i.v.	week 102
	Cytarabine	75 mg/m^2/d	i.v.	8 doses, weeks 102-103
	6-thioguanine	60 mg/m^2/d	p.o.	
	Triple i.t.	See above	i.t.	week 99
Maintenance 2	Methotrexate	20 mg/m^2/week	p.o	weekly
	6-mercaptopurine	75 mg/m^2/d	p.o.	daily
	Methotrexate	8-12 mg (by age)	i.t.	every 8 weeks

Granulocyte colony-stimulating factor (G-CSF) is given mainly after the first week, when the other therapeutics have been administered.

Supplementary table 2. The multivariate and univariate analyses of the event-free survival in the high-risk arms (chemotherapy and hSCT) of NOPHO ALL2008

		MULTIVARIATE			UNIVARIATE		
		**HR**	**95% CI**	***p***	**HR**	**95% CI**	***p***
	**N**						
**Sex**							
male	188	1*			1*		
female	126	1.25	0.75, 2.08	0.40	1.07	0.68, 1.68	0.76
**WBC (10^9/l)**							
<100	194	1*			1*		
≥100	120	2.80	1.42, 5.51	0.003	2.00	1.27, 3.17	0.003
**Lineage**							
B-ALL	185	1*			1*		
T-ALL	129	1.12	0.59, 2.16	0.72	1.35	0.86, 2.11	0.19
**Induction therapy**							
Prednisolone	122	1*			1*		
Dexamethasone	192	0.86	0.33, 2.23	0.75	2.00	1.12, 3.57	0.02
**Age (years)**							
1 - <10	140	1*			1*		
≥10 - <18	82	1.56	0.83, 2.92	0.16	1.17	0.66, 2.06	0.59
≥18	92	2.33	1.26, 4.30	0.007	1.71	1.01, 2.88	0.04
**Genetic subgroup**							
No high-risk aberrations	241	1*			1*		
KMT2A-rearrengement	54	0.64	0.28, 1.45	0.28	0.58	0.31, 1.11	0.10
Hypodiploid	19	1.94	0.71, 5.26	0.19	0.98	0.43, 2.28	0.97
**MRD at EOI**							
negative	22	1*			1*		
<0.1%	22	2.02	0.49, 8.33	0.33	2.59	0.65, 10.4	0.18
≥0.1% - <5%	143	2.58	0.69 , 9.65	0.16	3.60	1.12, 11.5	0.03
≥5%	87	3.52	0.89, 14.0	0.07	4.00	1.11, 14.4	0.03

Cox proportional-hazards regression calculated for known risk factors.

* marks reference groups of each categorical variable.

The PH assumption was evaluated (p>0.05) using the R's *survival* library's *cox.zph()* function.

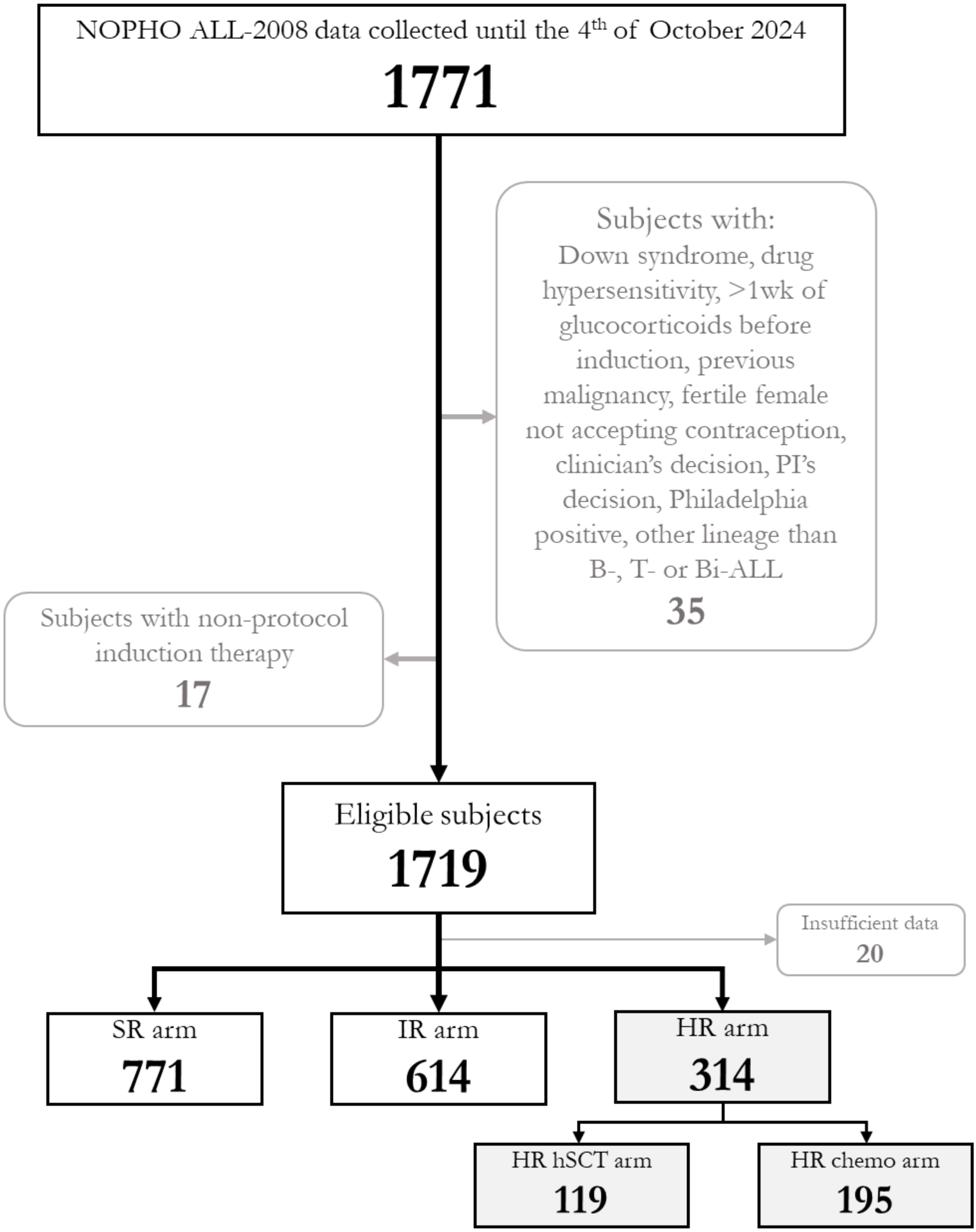

Supplementary figure 1. The flow chart for the selection process of participants and the exclusion criteria used in the NOPHO ALL2008 protocol.

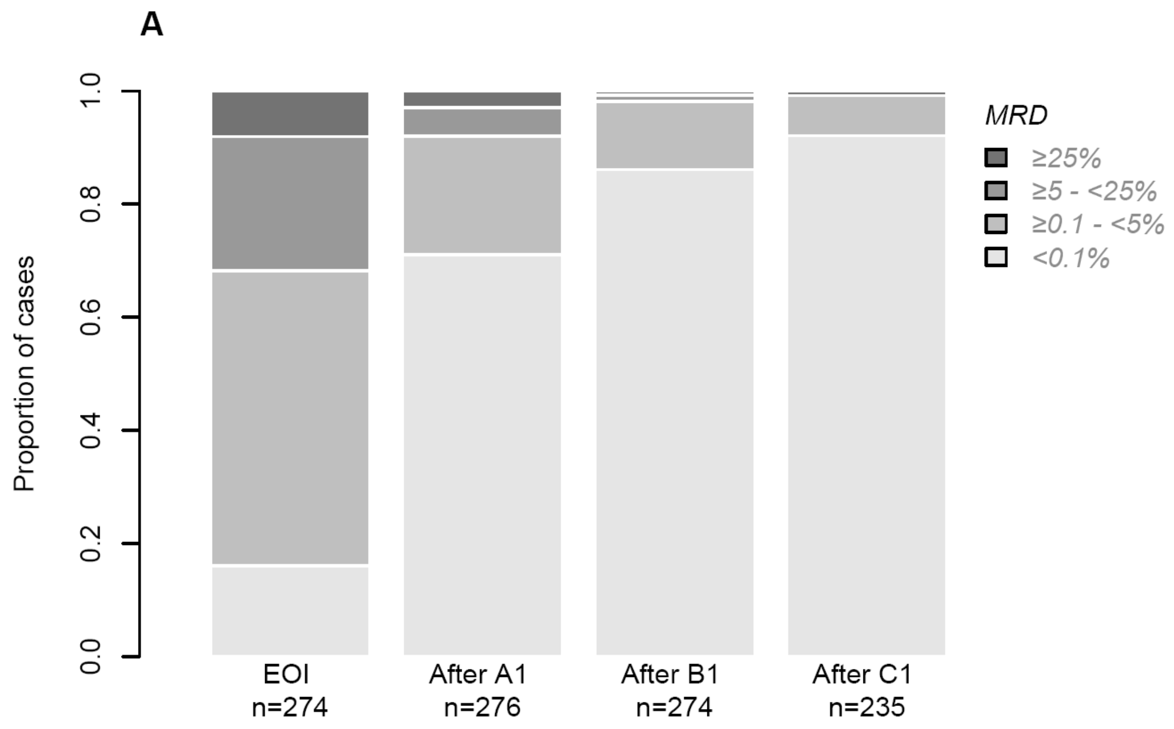

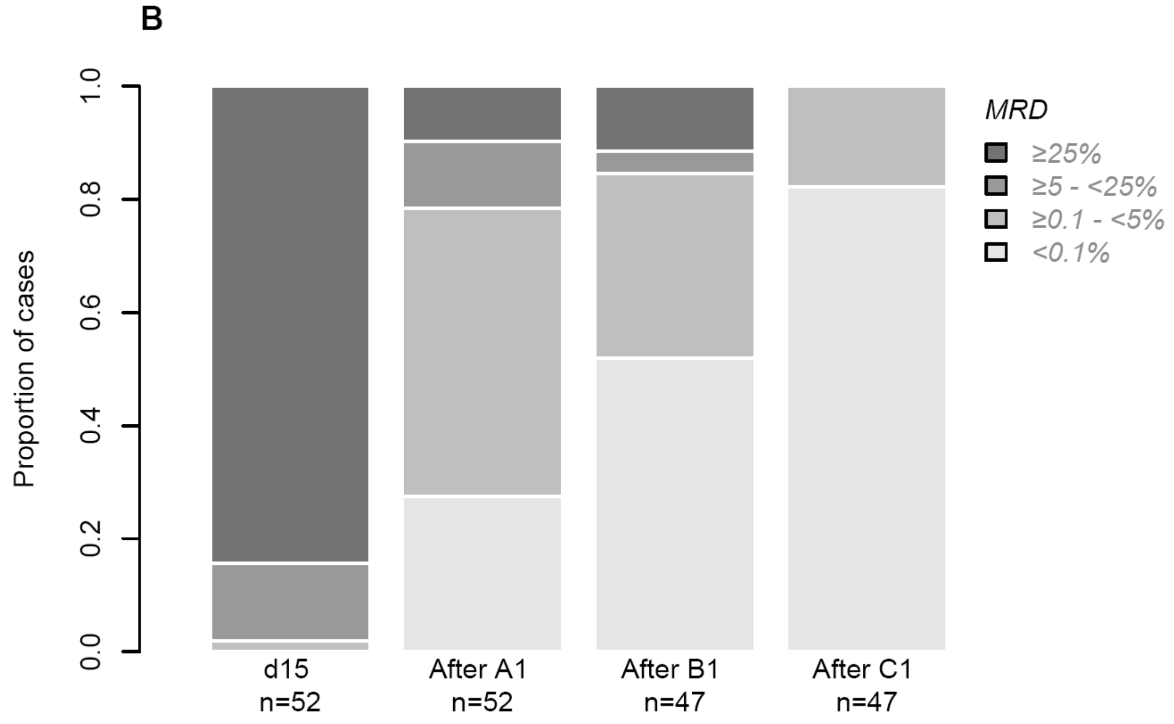

Supplementary figure 2. The therapy response at specified time points during the course of treatment. Proportions account for patients with available data for the particular analysis.

A: The entire HR cohort including HSCT patients.

B: Patients with slow early response, defined as >15% blasts bone marrow d15

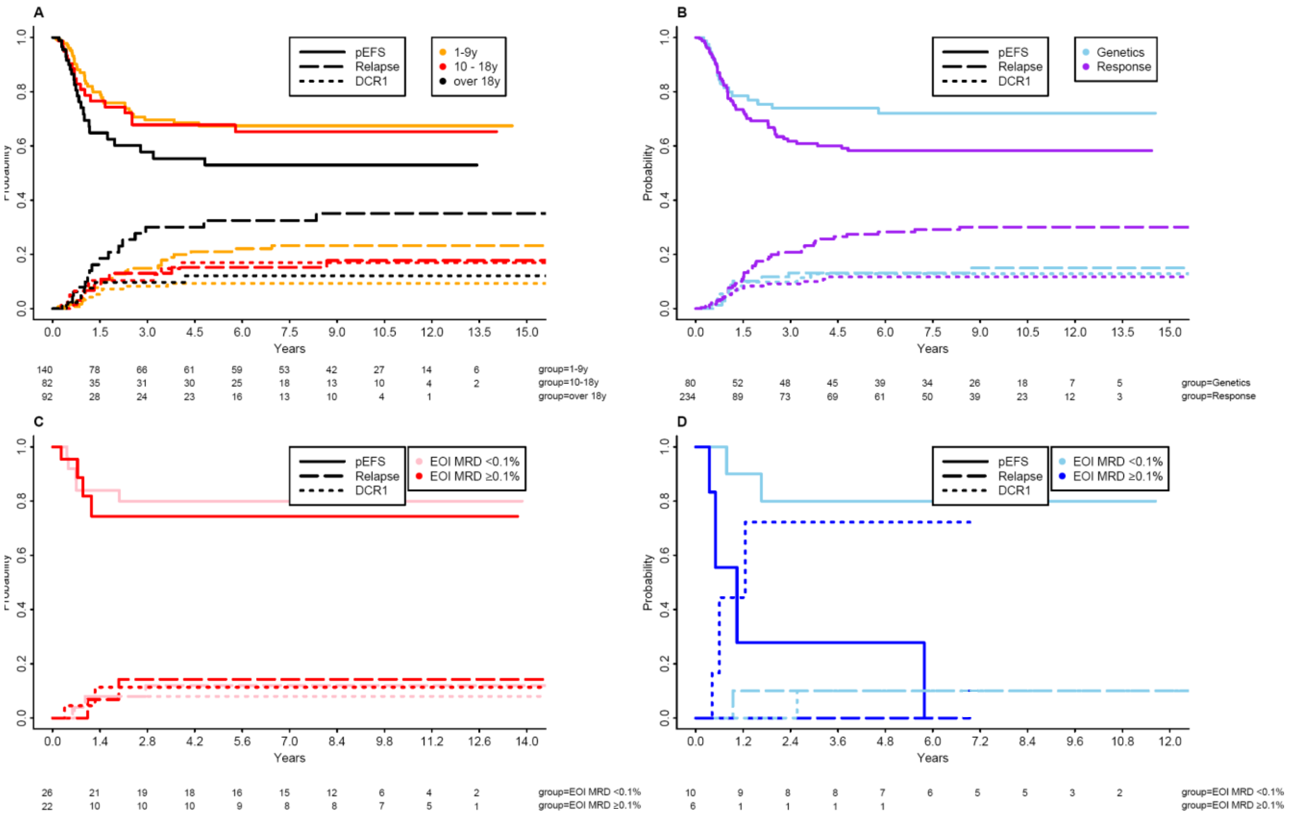

Supplementary figure 3. The event-free survival of HR chemotherapy patients, categorized by age groups, therapy response in genetic groups and stratification criteria. The number of patients at risk is provided at the bottom of each panel.

A: Event-free survival and cumulative incidence of death and relapse by age groups.

B: Event-free survival and cumulative incidence of death and relapse by stratification criteria.

C: Event-free survival and cumulative incidence of death and relapse of the *KMT2A*-r cohort by induction response.

D: Event-free survival and cumulative incidence of death and relapse of the hypodiploid cohort by induction response.
